# Proprioception in patients with posterior cruciate ligament tears: A meta-analysis comparison of reconstructed and contralateral normal knees

**DOI:** 10.1371/journal.pone.0184812

**Published:** 2017-09-18

**Authors:** Jung-Ro Yoon, Dae-Hee Lee, Seung-Nam Ko, Young-Soo Shin

**Affiliations:** 1 Department of Orthopedic Surgery, Veterans Health Service Medical Center, Seoul, Korea; 2 Department of Orthopedic Surgery, Samsung Medical Center, Sungkyunkwan University School of Medicine, Seoul, Korea; Universidad de Zaragoza, SPAIN

## Abstract

Posterior cruciate ligament (PCL) reconstruction for patients with PCL insufficiency has been associated with postoperative improvements in proprioceptive function due to mechanoreceptor regeneration. However, it is unclear whether reconstructed PCL or contralateral normal knees have better proprioceptive function outcomes. This meta-analysis was designed to compare the proprioceptive function of reconstructed PCL or contralateral normal knees in patients with PCL insufficiency. All studies that compared proprioceptive function, as assessed with threshold to detect passive movement (TTDPM) or joint position sense (JPS) in PCL reconstructed or contralateral normal knees were included. JPS was calculated by reproducing passive positioning (RPP). Five studies met the inclusion/exclusion criteria for the meta-analysis. The proprioceptive function, defined as TTDPM (95% CI: 0.25 to 0.51°; P<0.00001) and RPP (95% CI: 0.19 to 0.45°; P<0.00001), was significantly different between the reconstructed PCL and contralateral normal knees. The mean difference in angle of error between the reconstructed PCL and contralateral normal knees was 0.06° greater in TTDPM than by RPP. In addition, results from subgroup analyses, based on the starting angles and the moving directions of the knee, that evaluated TTDPM at 15° flexion to 45° extension, TTDPM at 45° flexion to 110° flexion, RPP in flexion, and RPP in extension demonstrated that mean angles of error were significantly greater, by 0.38° (P = 0.0001), 0.36° (P = 0.02), 0.36° (P<0.00001), and 0.23° (P = 0.04), respectively, in reconstructed PCL than in contralateral normal knees. The proprioceptive function of PCL reconstructed knees was decreased, compared with contralateral normal knees, as determined by both TTDPM and RPP. In addition, the amount of loss of proprioception was greater in TTDPM than in RPP, even with minute differences. Results from subgroup analysis, that evaluated the mean angles of error in moving directions through RPP, suggested that the moving direction of flexion has a significantly greater mean for angles of error than the moving direction of extension. Although the level of differences between various parameters were statistically significant, further studies are needed to determine whether the small differences (>1°) of the loss of proprioception are clinically relevant.

## Introduction

Proprioception includes the ability to detect passive movement (kinaesthesia) and the awareness of joint position (joint position sense) as well as the cumulative neural input to the central nervous system from mechanoreceptors, which consist of Pacinian corpuscles and Ruffini corpuscles. Pacinian corpuscles are stimulated during rapid changes in velocity and direction, whereas Ruffini corpuscles are slow-adapting and related to the joint position registration.[1–3] Injuries to the posterior cruciate ligament (PCL) may lead to impaired proprioceptive function, deterioration of position sense, and cartilage damage, and are associated with subsequently degenerative changes in long-term follow-up^1^. Therefore, PCL reconstruction for patients with PCL insufficiency is associated with proprioceptive function recovery through mechanoreceptor regeneration, with considerable afferent functions.[4,5] Although many studies on proprioception and mechanoreceptors of the anterior cruciate ligament (ACL) have been conducted,[6,7] few studies have assessed the PCL, and many results have been inconclusive because of the lower prevalence of PCL tears compared with ACL tears.[8,9] In addition, no systematic reviews or meta-analyses on this subject have been published.

Therefore, this meta-analysis compared the proprioceptive function of reconstructed PCL or contralateral normal knees in patients with PCL insufficiency by evaluating the threshold to detect passive movement (TTDPM) or reproducing passive positioning (RPP). We hypothesized that proprioceptive function would decrease more in reconstructed PCL than in contralateral normal knees.

## Materials and methods

This meta-analysis was conducted according to the guidelines of the preferred reporting items for systematic reviews and meta-analysis (PRISMA) statement (S1 PRISMA Checklist).

### Data and literature sources

This study followed the Cochrane Review Methods. Multiple comprehensive databases, including MEDLINE (January 1, 1976 to Dec 31, 2016), EMBASE (January 1, 1985 to Dec 31, 2016), Web of Science (January 1, 1980 to Dec 31, 2016), SCOPUS (January 1, 1980 to Dec 31, 2016), and the Cochrane Library (January 1, 1987 to Dec 31, 2016) were searched for studies that compared proprioceptive function as assessed with TTDPM or RPP between reconstructed PCL or contralateral normal knees. There were no restrictions on language. Search terms used in the title, abstract, MeSH, and keywords fields included (‘posterior cruciate ligament’ [Mesh] OR ‘PCL’ [tiab]) AND ‘proprioception’ [tiab] OR ‘threshold to detect passive movement’ [tiab] OR ‘reproducing passive positioning’ [tiab]). After the initial electronic search, relevant articles and their bibliographies were manually searched.

### Study selection

From the title and abstract, two reviewers independently selected the relevant studies for full review. The full text copy of the article was reviewed if the abstract did not provide enough data to make a decision. Studies were included in the meta-analysis if they (1) assessed human knees with a PCL tear followed by PCL reconstruction; (2) included study with level of evidence 1 to 3; (3) reported retrospective or prospective comparison of proprioceptive function between reconstructed PCL or contralateral normal knees; (4) included data on at least one of the following two parameters: TTDPM and/or RPP. For the TTDPM test, patients were asked to press a switch immediately on perception of sensation of motion of the knee during passive flexion or extension from a specific starting angle of the knee joint. The RPP test was assessed by passively moving the knee joint to a predetermined target angle. The patients were educated to remember that target position, and the knee was then returned to the starting positions. The patients were asked to press a switch when he or she felt that the angle of the knee joint had reached the target angle. Moreover, measurement of the TTDPM and RPP used a similar dynamometer apparatus in the included studies; (5) fully reported the number of subjects in each group (PCL reconstructed and contralateral normal knees) and the means and standard deviations for the two parameters; and (6) used adequate statistical methods to compare these parameters between groups. Studies were excluded if (1) they dealt with human knees with PCL tear without reconstruction or before reconstruction, (2) they did the use of techniques to measure proprioception other than TTDPM and/or RPP, (3) they did include missing or inadequate outcome data, such as standard deviation or range of values, or (4) they did include case series, expert opinions, reviews, commentaries, or editorials.

### Data extraction

Two reviewers independently recorded data from each study using a predefined data extraction form. Disagreement between the reviewers was resolved by consensus or by discussion with a third investigator when consensus could not be reached. Recorded variables included those associated with proprioceptive function between reconstructed PCL or contralateral normal knees. Sample size and the means and standard deviations of TTDPM and RPP in each group were also recorded. If a study presented different starting angles and moving directions of the knee for the TTDPM and RPP, data from different starting angles and moving directions were analyzed as separate studies. If these variables were not included in the articles, the study authors were contacted by email to retrieve further information. Authors of one study offered measured parameters such as means and standard deviations in response to our requests.

### Methodological quality assessment

Two reviewers independently assessed the methodological quality of the studies. For the Newcastle-Ottawa Scale,[10] as recommended by the Cochrane Non-Randomized Studies Methods Working Group, we assessed the studies based on three criteria: selection of the study groups, comparability of the groups, and ascertainment of either the exposure or the outcome of interest for case-control and cohort studies. Studies with scores ≥6 points were defined as high quality. Any unresolved disagreements between reviewers were resolved by consensus or by consultation with a third investigator. Publication bias could not be assessed in these trials. Tests for funnel plot asymmetry are typically performed only when at least ten studies are included in the meta-analysis.[11] As our analysis included only five studies, tests for asymmetry were not performed because these tests would not be able to differentiate asymmetry from chance.

### Data synthesis and analysis

The main outcomes of the meta-analysis were the standardized mean differences (SMDs) in TTDPM and RPP between reconstructed PCL and contralateral normal knees. For all comparisons, SMDs and 95% confidence intervals (CIs) were calculated for continuous outcomes. Heterogeneity was determined by estimating the proportion of between-study inconsistencies due to actual differences between studies, rather than differences due to random error or chance, using the I^2^ statistic, with values of 25%, 50%, and 75% considered as low, moderate, and high heterogeneity, respectively. All statistical analyses were performed with RevMan version 5.2 software. Subgroup analysis based on the starting angles and the moving directions of the knee was performed for the TTDPM and RPP in an attempt to explore a potential source of heterogeneity. In addition, starting angles and the moving directions of the knee have been considered in TTDPM and RPP measurements because different muscles, tendons, and ligaments can be most active and thus different amounts of mechanoreceptors can be active in particular directions even though TTDPM tests appear to be more consistent than JPS tests due to its relative simplicity. As a result, two subgroups were created in each group: 15° flexion to 45° extension and 45° flexion to 110° flexion for the TTDPM/ flexion and extension for the RPP. Moreover, sensitivity analysis was performed by excluding one of the eligible studies at a time; 2 studies with retrospective data were included. Pooling of data was feasible for only two outcomes of interest, i.e. TTDPM (15° flexion to 45° extension) and RPP (flexion).

## Results

### Study identification

**Fig 1:**
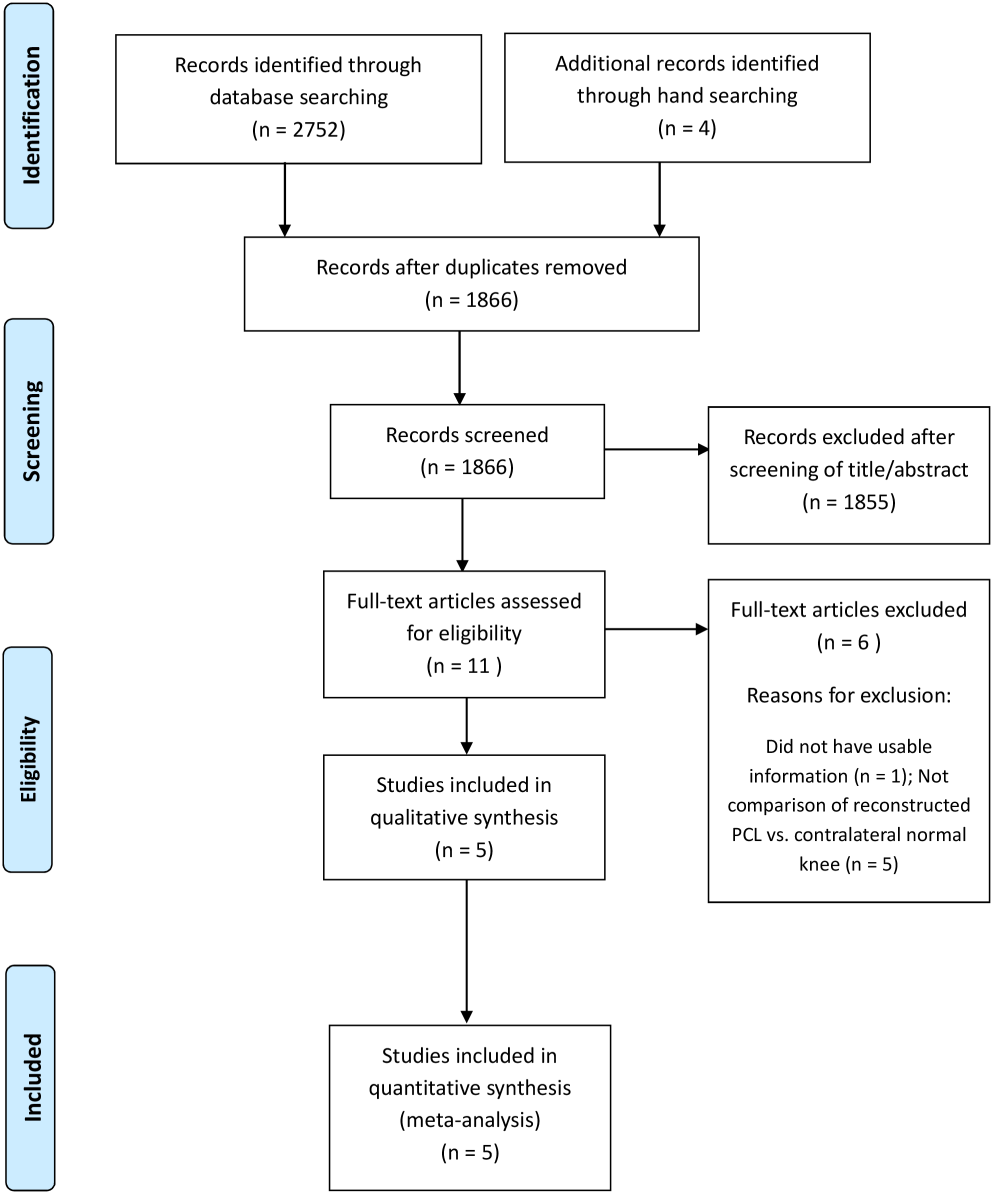
Preferred reporting items for systemic reviews and meta-analyses (PRISMA)flow diagram of literature selection.

Details on study identification, inclusion, and exclusion are summarized in Fig 1. An electronic search yielded 899 studies in PubMed (MEDLINE), 800 in EMBASE, 210 in Web of science, 821 in SCOPUS, and 22 in the Cochrane Library. Four additional publications were identified through manual searching. After removing 890 duplicates, 1866 studies remained; of these, 1855 were excluded based on abstract and full-text article review, and an additional six studies were excluded because they had unusable information or made inappropriate group comparisons. This eventually resulted in five studies that were included in the meta-analysis.[12–16]

### Study characteristics and patient populations

The five studies we examined included 241 subjects with reconstructed PCLs and contralateral normal knees that underwent proprioceptive function by TTDPM or RPP. Three studies compared prospectively measured parameters, whereas the other two studies compared parameters measured by retrospective chart review. Three studies compared both TTDPM and RPP, one compared TTDPM alone, and one compared RPP alone. Of the 4 studies that compared TTDPM, one compared TTDPM at a knee angle of both 15° and 45°, one compared TTDPM at a knee angle of both 20° and 45°, and two compared TTDPM at a knee angle of both 45° and 110°. Of the 4 studies that compared RPP, one compared RPP at a knee angle of both 15° and 45°, one compared RPP at a knee angle of both 0° and 90°, one compared RPP at a knee angle of both 45° and 110°, and one compared RPP at a knee angle of 90° (Table 1).

**Table 1:** Summary of patient characteristics of the included studies.

Study	Year	Study type	Sample size	Mean age (years)	Mean follow-up (Months)	Time from surgery to proprioception test (Months)	Measured parameters	Quality score
Adachi et al.[12]	2007	PCS	29	31.9	42	Mean 24	RPP (90°)	8
Lee et al.[13]	2013	RCS	20	36	61.3	At least 24	TTDPM (45°, 110°), RPP (45°, 110°)	7
Lee et al.[14]	2014	RCS	92	35.6	48.2	At least 24	TTDPM (20°, 45°), RPP (0°, 90°)	8
Li et al.[15]	2016	PCS	90	31.4	67.2	Mean 60	TTDPM (15°, 45°), RPP (15°, 45°)	8
Safran et al.[16]	1999	PCS	10	31	27	Mean 27	TTDPM (45°, 110°)	8

Abbreviations: PCS, prospective comparative study; RCS, retrospective comparative study; RPP, reproducing passive positioning; TTDPM, threshold to detect passive movement.

### Methodological quality assessment

The quality of the five studies included in the meta-analysis is summarized in Table 1. The non-RCTs (three PCSs and two RCSs) were of high quality (Newcastle-Ottawa Scale > 6).

### Threshold to detect passive movement (TTDPM)

**Fig 2:**
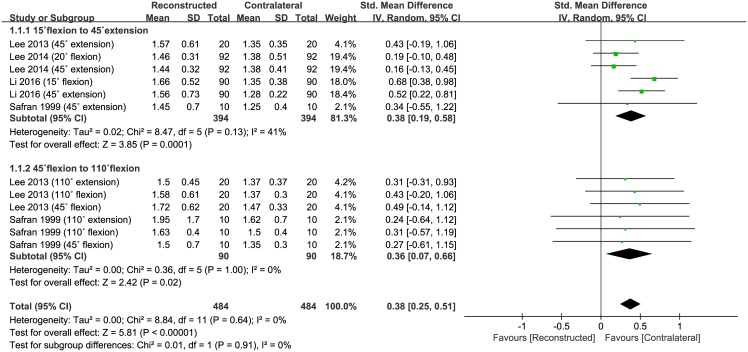
Results of aggregate analysis for comparison of threshold to detect passive movement (TTDPM) according to different modalities, including subgroup analysis based on the starting angles and the moving directions of the knee by 15° flexion to 45° extension and 45° flexion to 110° flexion.

Of the five studies, four compared TTDPM of reconstructed PCLs and contralateral normal knees, and included 484 subjects that had TTDPM performed in reconstructed PCL knees and 484 in contralateral normal knees. The pooled data showed that the mean TTDPM was 0.38° (95% CI: 0.25 to 0.51°; P<0.00001; I^2^ = 0%, Fig 2), indicating that TTDPM was significantly greater in reconstructed PCL than in contralateral normal knees. Five studies were assigned to the 15° flexion to 45° extension subgroup, and six studies were assigned to the 45° flexion to 110° flexion subgroup. The 15° flexion to 45° extension subgroup was associated with a mean angle of error that was significantly greater by 0.38° (95% CI: 0.19 to 0.58°; P = 0.0001; I^2^ = 41%, Fig 2) in the reconstructed PCL than in the contralateral normal knees. Similarly, 45° flexion to 110° flexion subgroup showed a mean angle of error that was significantly greater, by 0.36° (95% CI: 0.07 to 0.66°; P = 0.02; I^2^ = 0%, Fig 2) in the reconstructed PCL than in the contralateral normal knees. The results of sensitivity analysis were not significantly different from those of the original analysis, including that the findings are robust to the decisions made in the process of obtaining them (Table 2).

**Table 2:** Sensitivity analysis.

Study	Parameter	Before exclusion	After exclusion	Statistical significance
Lee et al.[13] (2013)	TTDPM (15° flexion to 45° extension)	SMD = 0.38, 95% CI = 0.19 to 0.58, Z = 3.85, P = 0.0001	SMD = 0.38, 95% CI = 0.16 to 0.60, Z = 3.36, P = 0.0008	No difference
	RPP (flexion)	SMD = 0.36, 95% CI = 0.21 to 0.52, Z = 4.51, P< 0.00001	SMD = 0.34, 95% CI = 0.17 to 0.51, Z = 3.92, P< 0.0001	No difference
Lee et al.[14] (2014)	TTDPM (15° flexion to 45° extension)	SMD = 0.38, 95% CI = 0.19 to 0.58, Z = 3.85, P = 0.0001	SMD = 0.57, 95% CI = 0.37 to 0.76, Z = 5.70, P< 0.00001	No difference
	RPP (flexion)	SMD = 0.36, 95% CI = 0.21 to 0.52, Z = 4.51, P< 0.00001	SMD = 0.41, 95% CI = 0.22 to 0.60, Z = 4.22, P< 0.0001	No difference

TTDPM, threshold to detect passive movement; RPP, reproducing passive positioning; SMD, standardized mean difference

### Reproducing passive positioning (RPP)

**Fig 3:**
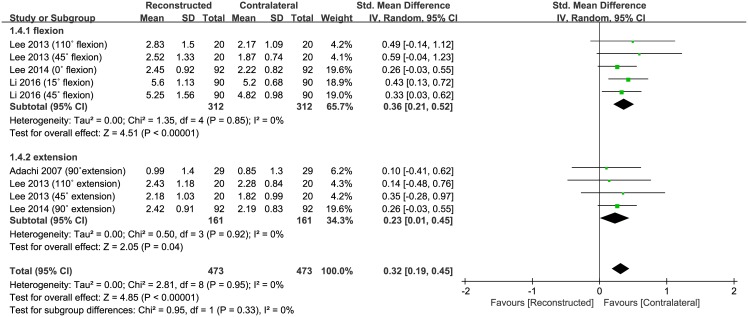
Results of aggregate analysis for comparison of reproducing passive positioning (RPP) according to different modalities, including subgroup analysis based on the moving directions of the knee by flexion and extension.

Of the five studies, four compared RPP of reconstructed PCL and contralateral normal knees, and included 473 subjects that had RPP performed in reconstructed PCL knees and 473 in contralateral normal knees. The pooled data showed that the mean RPP was 0.32° (95% CI: 0.19 to 0.45°; P<0.00001; I^2^ = 0%, Fig 3), indicating that RPP was significantly greater with PCL reconstruction than in contralateral normal knees. Five studies were assigned to the flexion subgroup, and four studies were assigned to the extension subgroup. The flexion subgroup showed mean angle of error that was 0.36° greater (95% CI: 0.21 to 0.52°; P<0.00001; I^2^ = 0%, Fig 3) in the reconstructed PCL than in the contralateral normal knees. Similarly, the extension subgroup indicated a mean angle of error that was 0.23° greater (95% CI: 0.01 to 0.45°; P = 0.04; I^2^ = 0%, Fig 3) in the reconstructed PCL than in the contralateral normal knees. The results of sensitivity analysis were not significantly different from those of the original analysis, including that the findings are robust to the decisions made in the process of obtaining them (Table 2).

## Discussion

The most important finding of this meta-analysis was that proprioceptive function of reconstructed PCL knees was decreased compared with contralateral normal knees, as determined by both TTDPM and RPP. In addition, the amount of loss of proprioceptive function was greater in TTDPM than in RPP, even with minute differences.

This meta-analysis showed that both TTDPM (0.38°) and RPP (0.32°) were significantly greater with PCL reconstruction than in contralateral normal knees, even with minute differences which are unlikely to be clinically relevant.[17] A proprioceptive deficit of more than 5° is considered to be clinically relevant.[18] It is possible that the PCL reconstructed knees did not have a proprioception deficit large enough to affect functional declines because of PCL reconstruction with remnant preservation and the fact that the duration from surgery to proprioception test was longer than 24 months, resulting in recovering proprioceptive function gradually, similar to findings in knee with ACL reconstruction.[19] Preserving the remnant PCL fibers may help restore sensation for joint motion and position by saving mechanoreceptors, which can lead to proprioceptive function recovery to the level of the contralateral normal knees.[20] These results were consistent with those of the Eguchi *et al*. study that evaluated the TTDPM between preoperative assessment and any follow-up point and did not find any significant differences between PCL reconstructed knees with remnant preservation and contralateral normal knees, regardless of the starting angles and the moving directions of the knees.[9] This result was similar to previous findings that showed that remnant preserving PCL reconstruction was effective in restoring proprioception, such as TTDPM and RPP, to the level of the contralateral normal knees.[13] Another factor that can explain these results may be a loss of proprioception in the contralateral normal knees, suggesting that the altered afferent information from the articular mechanoreceptors, such as Pacinian corpuscles and Ruffini corpuscles in the PCL reconstructed knees, may contribute to and alter the sense of stability in the contralateral normal knees, which could impact the number of proprioceptive deficits than in healthy subjects.[6,21]

Previous findings have indicated that there are no available valid measurement tools for detecting the clinically significant importance of proprioceptive function.[22] For example, most joint position sense (JPS) findings could be attributable to a lack of reliability and validity because of relatively complicated measurements caused by various confounding factors such as starting angle, direction of movement, target angle, and angular velocity.[23,24] In addition, TTDPM detects the low threshold response and rapid-adapting mechanoreceptors, such as Pacinian corpuscles, which may make it more sensitive than JPS, which has a low threshold but slow-adapting mechanoreceptors such as Ruffini corpuscles.[25,26] Therefore, TTDPM may not completely detect the real differences, resulting in a lower magnitude of change compared to JPS. In contrast with our expectations, the current meta-analysis found that the mean difference in angle of error between reconstructed PCL and contralateral normal knees was 0.06° greater by TTDPM than by RPP, suggesting that the TTDPM results are less constant than for JPS. These results may be attributable to the fact that information from other sensory pathways in patients with TTDPM may have unexpectedly influenced the mean angle of error. For example, previous studies found conflicting results, including a reported mean angle of error of 2.4° for elderly patients after knee arthroplasty and 2.7° for young dancers.[27,28]

Many studies reported that healthy subjects with JPS measurements had greater mean angles of error in the moving direction of flexion than the moving direction of extension. These results can be explained by the fact that the moving direction of flexion may offer fewer levels of afferent feedback because of less muscle spindle activation in the smaller hamstring muscle group, compared with the larger quadriceps muscle group contraction during the moving direction of extension.[29,30] Indeed, our subgroup analysis findings that evaluated mean angles of error in the moving directions through RPP, suggested that the moving direction of flexion (0.36°) is significantly greater for mean angles of error than the moving direction of extension (0.23°).

This study had several limitations. All five studies were observational, which caused some inherent heterogeneity due to uncontrolled bias, even though the studies had high quality scores. In addition, the heterogeneity of the included studies could be explained by slight differences in other factors affecting proprioceptive function, including the use of a wide variety of rehabilitation programs and variability in directions of movement and starting angles. Finally, proprioception during dynamic activities or weight-bearing was not evaluated.

## Conclusions

The proprioceptive function of PCL reconstructed knees was decreased compared with contralateral normal knees, as determined by both TTDPM and RPP. In addition, the amount of loss of proprioception was greater in TTDPM than in RPP, even with minute differences. Results from subgroup analysis that evaluated mean angles of error in terms of the moving directions through RPP suggested that the moving direction of flexion is significantly greater in mean angles of error than the moving direction of extension.

## Supporting information

S1 PRISMA Checklist PRISMA checklist. (DOC)

## References

[1] Clark P, MacDonald PB, Sutherland K. Analysis of proprioception in the posterior cruciate ligament-deficient knee. Knee Surg Sports Traumatol Arthrosc 1996;4(4):225–227. 9046507 10.1007/BF01567967

[2] Lephart SM, Pincivero DM, Giraldo JL, Fu FH. The role of proprioception in the management and rehabilitation of athletic injuries. Am J Sports Med. 1997;25(1):130–137. doi: 10.1177/036354659702500126 9006708 10.1177/036354659702500126

[3] Raunest J, Sager M, Burgener E. Proprioception of the cruciate ligaments: receptor mapping in an animal model. Arch Orthop Trauma Surg. 1998;118(3):159–163. 9932192 10.1007/s004020050338

[4] Jung HJ, Kim JH, Lee HJ, Koo s, Chang SH, Jung YB, et al The isometry of two different paths for remnant-preserving posterior cruciate ligament reconstruction. Knee Surg Sports Traumatol Arthrosc. 2013;21(5):1029–1035. doi: 10.1007/s00167-012-2111-6 22763568 10.1007/s00167-012-2111-6

[5] Liabaud B, Patrick DA, Geller JA. Is the posterior cruciate ligament destabilized after the tibial cut in a cruciate retaining total knee replacement?: An anatomical study. Knee. 2013;20(6):412–415. doi: 10.1016/j.knee.2013.02.001 23566736 10.1016/j.knee.2013.02.001

[6] Arockiaraj J, Korula RJ, Oommen AT, Devasahayam S, Wankhar S, Velkumar S, et al Proprioceptive changes in the contralateral knee joint following anterior cruciate injury. Bone Joint J. 2013;95-B(2):188–191. doi: 10.1302/0301-620X.95B2.30566 23365027 10.1302/0301-620X.95B2.30566

[7] Katayama M, Higuchi H, Kimura M, Kobayashi A, Hatayama K, Terauchi M, et al Proprioception and performance after anterior cruciate ligament rupture. Int Orthop. 2004;28(5):278–281. doi: 10.1007/s00264-004-0583-9 15338203 10.1007/s00264-004-0583-9 PMC3456983

[8] Chouteau J, Testa R, Viste A, Moyen B. Knee rotational laxity and proprioceptive function 2 years after partial ACL reconstruction. Knee Surg Sports Traumatol Arthrosc. 2012;20(4):762–766. doi: 10.1007/s00167-012-1879-8 22258650 10.1007/s00167-012-1879-8

[9] Eguchi A, Adachi N, Nakamae A, Usman MA, Deie M, Ochi M. Proprioceptive function after isolated single-bundle posterior cruciate ligament reconstructionwith remnant preservation for chronic posterior cruciate ligament injuries. Orthop Traumatol Surg Res. 2014;100(3):303–308. doi: 10.1016/j.otsr.2013.12.020 24679366 10.1016/j.otsr.2013.12.020

[10] Wells GA, Shea B, O’connell D, Peterson J, Welch V, Losos M, et al. The Newcastle-Ottawa Scale (NOS) for assessing the quality of nonrandomised studies in meta-analyses. http://www.ohri.ca/programs/clinical_epidemiology/oxford.asp. Accessed Jul 1 2016.

[11] Deeks JJ, Higgins JPT, Altman DG, Green S. Cochrane handbook for systematic reviews of interventions version 5.1. 0 (Updated March 2011). The Cochrane Collaboration (2011).

[12] Adachi N, Ochi M, Uchio Y, Iwasa J, Ishikawa M, Shinomiya R. Temporal change of joint position sense after posterior cruciate ligament reconstruction using multi-stranded hamstring tendons. Knee Surg Sports Traumatol Arthrosc. 2007;15(1):2–8. doi: 10.1007/s00167-006-0127-5 16799825 10.1007/s00167-006-0127-5

[13] Lee DC, Shon OJ, Kwack BH, Lee SJ. Proprioception and clinical results of anterolateral single-bundle posterior cruciate ligament reconstruction with remnantpreservation. Knee Surg Relat Res. 2013;25(3):126–132. doi: 10.5792/ksrr.2013.25.3.126 24032101 10.5792/ksrr.2013.25.3.126 PMC3767898

[14] Lee DW, Jang HW, Lee YS, Oh SJ, Kim JY, Song HE, et al Clinical, Functional, and Morphological Evaluations of Posterior Cruciate Ligament Reconstruction With Remnant Preservation: Minimum 2-Year Follow-up. Am J Sports Med. 2014;42(8):1822–1831. doi: 10.1177/0363546514536680 24944294 10.1177/0363546514536680

[15] Li J, Kong F, Gao X, Shen Y, Gao S. Prospective Randomized Comparison ofKnee Stability and Proprioception for Posterior Cruciate Ligament Reconstruction With Autograft, Hybrid Graft, and gamma-Irradiated Allograft. Arthroscopy. 10.1016/j.arthro.2016.04.024 27282110

[16] Safran MH, Harrier CD, Giraldo JL. Effects of injury and Reconstruction of the Posterior Cruciate Ligament onroprioception and Neuromuscular Control.J Sports Rehab. 1999;8:304–321.

[17] Ogard WK. Proprioception in sports medicine and athletic conditioning. Strength & Conditioning Journal. 2011;33(3):111–118.

[18] Callaghan MJ, Selfe J, Bagley PJ, Oldham JA. The Effects of Patellar Taping on Knee Joint Proprioception. J Athl Train. 2002;37(1):19–24. 12937439 PMC164303

[19] Iwasa J, Ochi M, Adachi N, Tobita M, Katsube K, Uchio Y. Proprioceptive improvement in knees with anterior cruciate ligament reconstruction. Clin Orthop Relat Res 2000;381:168–176. 10.1097/00003086-200012000-00020 11127653

[20] Kim YM, Lee CA, Matava MJ. Clinical results of arthroscopic single-bundle transtibial posterior cruciate ligament reconstruction: a systematic review. Am J Sports Med. 2011;39(2):425–434. doi: 10.1177/0363546510374452 20702860 10.1177/0363546510374452

[21] Roberts D, Friden T, Stomberg A, Lindstrand A, Moritz U. Bilateral proprioceptive defects in patients with a unilateral anterior cruciate ligament reconstruction: a comparison between patients and healthy individuals. J Orthop Res. 2000;18(4):565–571. doi: 10.1002/jor.1100180408 11052492 10.1002/jor.1100180408

[22] Beynnon B, Renstrom P, Konradsen L, Elmqvist L, Gottlieb D, Dirks M. Validation of techniques to measure knee proprioception. Proprioception and neuromuscular control in joint stability. 2000:127–138.

[23] Grob K, Kuster M, Higgins S, Lloyd D, Yata H. Lack of correlation between different measurements of proprioception in the knee. J Bone Joint Surg Br. 2002;84(4):614–618. 12043789 10.1302/0301-620x.84b4.11241

[24] Herrington L. Knee-joint position sense: the relationship between open and closed kinetic chain tests. J Sport Rehab. 2005;14(4):356.

[25] Friden T, Roberts D, Zatterstrom R, Lindstrand A, Moritz U. Proprioception after an acute knee ligament injury: a longitudinal study on 16 consecutive patients. J Orthop Res. 1997;15(5):637–644. doi: 10.1002/jor.1100150502 9420590 10.1002/jor.1100150502

[26] Katonis PG, Assimakopoulos AP, Agapitos MV, Exarchou EI. Mechanoreceptorsin the posterior cruciate ligament. Histologic study on cadaver knees. Acta Orthop Scand. 1991;62(3):276–278. 2042472 10.3109/17453679108993609

[27] Barrack RL, Skinner HB, Brunet ME, Cook SD. Joint kinesthesia in the highlytrained knee. J Sports Med Phys Fitness. 1984;24(1):18–20. 6471834

[28] Cash RM, Gonzalez MH, Garst J, Barmada R, Stern SH. Proprioception after arthroplasty: role of the posterior cruciate ligament. Clin Orthop Relat Res. 1996;331(331):172–178. 8895635

[29] Matthews PB. Proprioceptors and their contribution to somatosensory mapping; complex messages require complex processing. Can J Physiol Pharmacol. 1988;66(4):430–438. 3048612 10.1139/y88-073

[30] Ribot-Ciscar E, Roll JP. Ago-antagonist muscle spindle inputs contribute together to joint movement coding in man. Brain Res. 1998;791(1–2):167–176. 9593876 10.1016/s0006-8993(98)00092-4

